# Development and Field Deployment of a Compact Dual-Range Infrared Carbon Dioxide Sensor System

**DOI:** 10.3390/s25051445

**Published:** 2025-02-27

**Authors:** Xiaoteng Liu, Xuehua Xiao, Zhening Zhang, Fang Song, Yiding Wang, Chuantao Zheng

**Affiliations:** State Key Laboratory of Integrated Optoelectronics, College of Electronic Science and Engineering, Jilin University, 2699 Qianjin Street, Changchun 130012, China; xiaoteng22@mails.jlu.edu.cn (X.L.); xiaoxh22@mails.jlu.edu.cn (X.X.); zzn22@mails.jlu.edu.cn (Z.Z.); songfang@jlu.edu.cn (F.S.); ydwang@jlu.edu.cn (Y.W.)

**Keywords:** dual-range, carbon dioxide sensor, dual-channel signal conditioning, multivariate linear regression, temperature and humidity compensation

## Abstract

A dual-range mid-infrared carbon dioxide (CO_2_) sensor is developed with temperature and humidity compensation functionalities. Using the same optical path, the sensor employs dual-channel signal processing circuits to achieve measurements across two detection ranges of 200–3000 parts-per-million (ppm) (low concentration range) and 8–25% (high concentration range), respectively. The developed sensor, with a compact size of 8.5 × 5.5 × 3.5 cm^3^, shows a good linear response, with fitting goodness *R*^2^ = 0.99942 for the low range and *R*^2^ = 0.9993 for the high range. Under environmental conditions of 20 °C temperature and 30% relative humidity and with an averaging time of 1 s, the limits of detection are 0.15 ppm for the low range and 32.4 ppm for the high range, respectively. A temperature and humidity compensation scheme based on multiple linear regression is proposed to mitigate the impact of environmental temperature and humidity changes. The experimental results demonstrate that the relative error after compensation is reduced from 21% to 0.87%. Indoor and outdoor CO_2_ measurements are performed to validate the good characteristics of the sensor system.

## 1. Introduction

The concentration of carbon dioxide (CO_2_) has experienced rapid escalation, notably during the industrialization period [1,2,3,4]. Since 1700, the global average CO_2_ concentration has risen from 280 parts-per-million (ppm) before the industrial revolution to 424 ppm at present [5,6], and the global average temperature has increased by 1.1 °C since 1880 [7]. The surge in CO_2_ concentration is one of the main causes of global warming and climate change [8,9]. According to the Occupational Safety and Health Administration (OSHA), the permissible exposure limit (PEL) for carbon dioxide is established as a 10,000 ppm 8 h time-weighted average (TWA) and a 30,000 ppm short-term exposure limit (STEL). Thus, it becomes crucial to monitor CO_2_ concentration and control CO_2_ emissions. This invokes a high demand on the performance of CO_2_ sensors [10,11].

CO_2_ sensors based on the non-dispersive infrared (NDIR) principle offer the advantages of low cost, excellent stability, long lifespan, and wide measurement range [12,13,14,15,16,17]. These benefits make the sensors suitable for diverse applications, including atmospheric environment monitoring, industrial production processes, and industrial exhaust emissions [18,19,20,21]. For example, in 2020, Trieu-Vuong Dinh et al. introduced an NDIR sensor featuring a wide measurement range through the utilization of dual optical paths [22]. In 2023, Han et al. developed an NDIR sensor for early thermal runaway warning in automotive batteries capable of detecting changes in CO_2_ concentration 25 s before battery failure [23]. In 2024, Xu et al. proposed a compact CO_2_ sensor designed for harsh environments, offering measurement capabilities for CO_2_ gas concentration levels ranging from 0.5% to 20% [24].

The NDIR CO_2_ sensors reported thus far are susceptible to interference from environmental factors such as temperature and humidity [25], leading to detection errors caused by changes in temperature and humidity, as well as a small measurement range, which limits their applications. To address these issues, we adopt a temperature and humidity compensation method based on multiple linear regression to rectify errors. Traditional dual-range sensors are implemented by changing their optical paths. Such a method complicates the mechanical structure and generates additional errors. Our approach is to adopt the same optical path while subtly processing the dual-channel signals, i.e., adjusting the processing channel and the measurement range to meet different application requirements. This approach not only simplifies the sensor structure but also leads to reduced errors and costs.

## 2. Design of the Compact Dual-Range CO_2_ Sensor System

### 2.1. Detection Principle

The essence of NDIR CO_2_ measurement lies in the absorption of infrared light with particular wavelengths by CO_2_ molecules. As the infrared light with wavelength *λ* and intensity *I*_0_ passes the detection channel with an optical path length *L*, the output intensity is weakened due to CO_2_ absorption and follows a specific attenuation pattern with the gas concentration *C*, which is expressed by the Beer–Lambert law as

(1) $$I=I_{0}\exp\left[-\delta (\lambda )CL\right]$$

where *I*_0_ indicates the input intensity, *I* is the output intensity after the light is absorbed by the gas, *δ* denotes the absorption coefficient of CO_2_ at wavelength *λ*, and *C* stands for gas concentration. This absorption characteristic can be utilized to quantitatively measure gas concentration. By measuring the change in the infrared light intensity before and after gas absorption, an accurate measurement of gas concentration can be achieved based on the calibrated sensor response between output light intensity and concentration.

### 2.2. Optical Path Design

**Figure 1:**
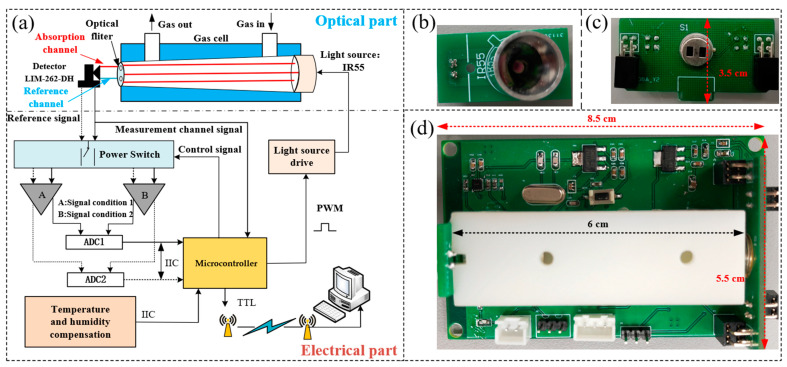
(**a**) Block diagram of the NDIR-based CO_2_ sensor system. (**b**) Light source IR55. (**c**) Detector LIM-262-DH. (**d**) Microcontroller circuit and gas cell.

A structural block diagram of the sensor system is depicted in Figure 1, primarily comprising a microcontroller (MCU, model: STM32F103C8T6, STMicroelectronics, Geneva, Switzerland, SZLCSC), a broadband thermal light source (Model: IR-55, Hawkeye Technologies, Milford, CT, USA, Alibaba Group), a dual-channel pyroelectric infrared detector (Model: LIM-262-DH,Pyroelectric infrared sensor, Dresden, Germany, Alibaba Group), a gas chamber, a temperature and humidity sensor (Model: SHT-31, Sensirion, Zürich, Switzerland, SZLCSC), a voltage-controlled current source circuit (Model: ADP7105, ADI, Wilmington, MA, USA, SZLCSC), an analog switch (Model: TPS2042,Texas Instruments, Dallas, TX, USA, SZLCSC), an analog-to-digital convertor (Model: LTC1864, ADI, Wilmington, MA, USA), and an adjustable gain operational amplifier circuit (Model: AD8620, ADI, Wilmington, MA, USA).

**Figure 2:**
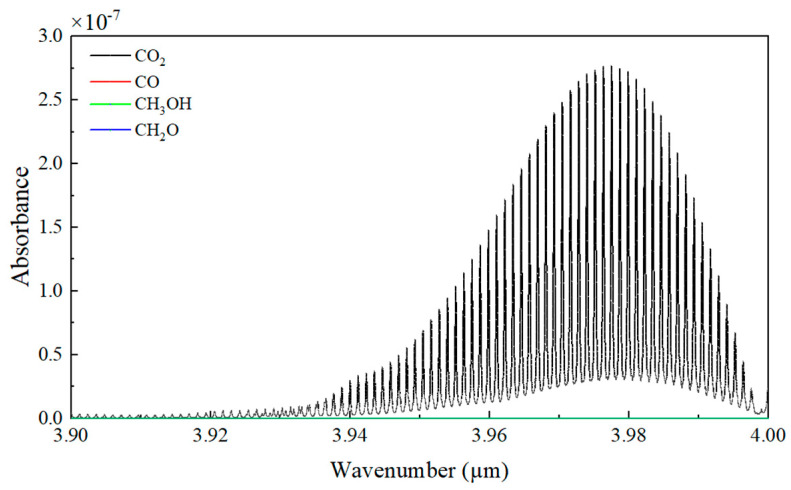
The absorbance of CO_2_, CO, CH_3_OH, and CH_2_O was simulated under the conditions of a temperature of 300 K, a pressure of 1 atm, and an optical path length of 1 cm. Because the absorption coefficients of CO, CH_3_OH and CH_2_O are too low, close to 0, they are not obvious in the figure.

The sensor employs a dual-channel differential detection structure with an optical path length of 4.8 cm. Channel 1 (A: Signal condition 1) is the absorption channel, while channel 2 (B: Signal condition 2) is the reference channel. The introduction of the reference channel is to eliminate the influence of light intensity fluctuations. Both channels have optical filters. The center wavelength of the absorption channel filter is *λ*_1_ = 4.26 μm (with a full width at half maximum of ±90 nm), corresponding to the strong absorption peak of CO_2_. The photoelectric conversion constant of the absorption channel is *k*_1_. The center wavelength of the reference channel filter is *λ*_2_ = 3.95 μm, and there is no absorption of CO_2_ and other gases in this wavelength range. As shown in Figure 2, CO, CH_3_OH, and CH_2_O exhibit no absorption at 3.95 μm. The photoelectric conversion constant of the reference channel is *k*_2_.

The light source is modulated electro-optically. The dual-channel detector collects the optical signal and converts it into analog voltage signals *U*_10_ and *U*_20_. The signal conditioning channel is adjusted according to the output voltage signal *U*_10_ of the detection channel. The microcontroller performs software phase-locking on the conditioned signals, obtains the characteristic parameters of the CO_2_ concentration, i.e., the amplitude of the first harmonic, and then transmits the characteristic parameters to a host computer for the inversion processing of the concentration.

### 2.3. Signal Conditioner

The amplitude of the output voltage signal, *U*_10_, of the detector is determined by gas concentration. According to the Beer–Lambert law, the higher the gas concentration, the stronger the attenuation of infrared light and the smaller the output voltage *U*_10_. Therefore, gas concentration can be determined by the output voltage signal from the detector, and on this basis, different signal conditioning channels can be selected to achieve gas concentration detection of different orders of magnitude.

**Figure 3:**
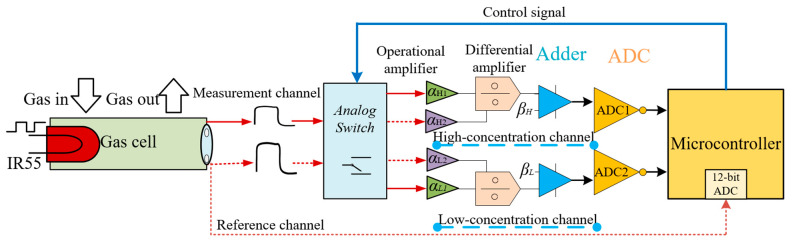
Schematic diagram of signal conditioning circuit. The subscripts “*H*” and “*L*” are the parameters for the high-concentration detection channel and low-concentration detection range, respectively.

The designed signal conditioner of the system is shown in Figure 3. Here, we define 200–3000 ppm as the low concentration range and 8–25% as the high concentration range. The output voltage signal *U*_10_ is split into two paths, one of which is collected by the 12-bit ADC built into the microcontroller. If the amplitude *U*_10_ is higher than the set threshold, which means the target gas concentration is in the range of 8–25%, the microcontroller controls the dual-channel voltage switch to switch *U*_10_ to the high-concentration signal conditioning channel. Otherwise, *U*_10_ is switched to the low-concentration signal conditioning channel. In this way, the measurement range is altered without changing the optical path, thus achieving dual-range measurement capability.

The voltage signals *U*_10_ and *U*_20_ are amplified by the dual-channel operational amplifier AD8620 to produce *U*_1_ and *U*_2_ as

(2) $$U_{1}=\alpha_{1}U_{10}=\alpha_{1}k_{1}(\lambda_{1})I(\lambda_{1})=\alpha_{1}k_{1}(\lambda_{1})I_{0}(\lambda_{1})\exp[-\delta (\lambda_{1})CL]$$

(3) $$U_{2}=\alpha_{2}U_{20}=\alpha_{2}k_{2}(\lambda_{2})I(\lambda_{2})=\alpha_{2}k_{2}(\lambda_{2})I_{0}(\lambda_{2})$$

In Equations (2) and (3), *α*_1_ and *α*_2_ represent the voltage signal amplification factors for the absorption channel and the reference channel, respectively, and the two parameters are adjustable. For different sensor measurement ranges, one should first introduce a gas sample with the minimum concentration of the range and adjust the gain *α*_2_ of the reference channel. If the ADC1 and ADC2 (Model LTC1864, ADI, Wilmington, MA, USA) sampling range is 5 V, the peak-to-peak value of *U*_2_ is between −2.5 V and 2.5 V. Then, adjust the gain *α*_1_ of the absorption channel to make *U*_1_ slightly less than *U*_2_, ensuring that the infrared light attenuated by the gas absorption is minimized and resulting in a maximum *U*_1_. This ensures that the differential signal Δ*U* = *U*_2_ − *U*_1_ remains positive throughout the measurement range. Subsequently, another gas sample is introduced with the maximum concentration of the range. At this point, the infrared light attenuated by the gas absorption is maximized, resulting in the maximum *U*_1_, and the differential signal Δ*U* is also maximized. The differential signal then enters the differential amplifier, where one adjusts the gain, *α*_3_, so that the peak-to-peak value of the output voltage *U*_3_ also remains within −2.5 V–2.5 V. This ensures that the output signal within the range does not exceed the ADC acquisition range. The value *U*_3_ can be expressed as

(4) $$U_{3}=U_{2}-U_{1}=\alpha_{3}(\alpha_{2}k(\lambda_{2})I_{0}(\lambda_{2})-\alpha_{1}k(\lambda_{1})I_{0}(\lambda_{1})\exp[-\delta (\lambda_{1})CL])$$

Since the detector operates with a dual power supply with a range of −5–5 V, the output signal may contain negative voltage signals that cannot be sampled by the ADC. To ensure complete signal sampling and enable the ADC to operate at full scale for improved detection accuracy, it is necessary to superimpose a positive bias voltage signal of *β* = 2.5 V to *U*_3_. The voltage signal after the addition of the bias voltage is denoted as *U*_4_:

(5) $$U_{4}=U_{3}+\beta =\left[\alpha_{3}(\alpha_{2}k(\lambda_{2})I_{0}(\lambda_{2})+\beta \right]-\alpha_{1}k(\lambda_{1})I_{0}(\lambda_{1})\exp[-\delta (\lambda_{1})CL])$$

Define

(6) $$U=\frac{U_{4}}{U_{2}}=\frac{\alpha_{3}\alpha_{2}k(\lambda_{2})I_{0}(\lambda_{2})+\beta }{\alpha_{2}k(\lambda_{2})I_{0}(\lambda_{2})}-\frac{\alpha_{1}k(\lambda_{1})I_{0}(\lambda_{1})}{\alpha_{2}k(\lambda_{2})I_{0}(\lambda_{2})}\exp[-\delta (\lambda_{1})CL]$$

where $m=\alpha_{3}(\alpha_{2}k(\lambda_{2})I_{0}(\lambda_{2})+\beta$, $n=\alpha_{1}k(\lambda_{1})I_{0}(\lambda_{1})$,$q=\delta (\lambda_{1})L$. Thus, Equation (6) can be simplified to

(7) U=m−nexp⁡[−qC]

From Equation (7), we derive the gas concentration based on the Beer–Lambert law as

(8) $$C=-\frac{1}{q}\ln(\frac{m-U}{n})$$

## 3. Performance Evaluation of the CO_2_ Sensor System

### 3.1. Calibration Experiment

**Figure 4:**
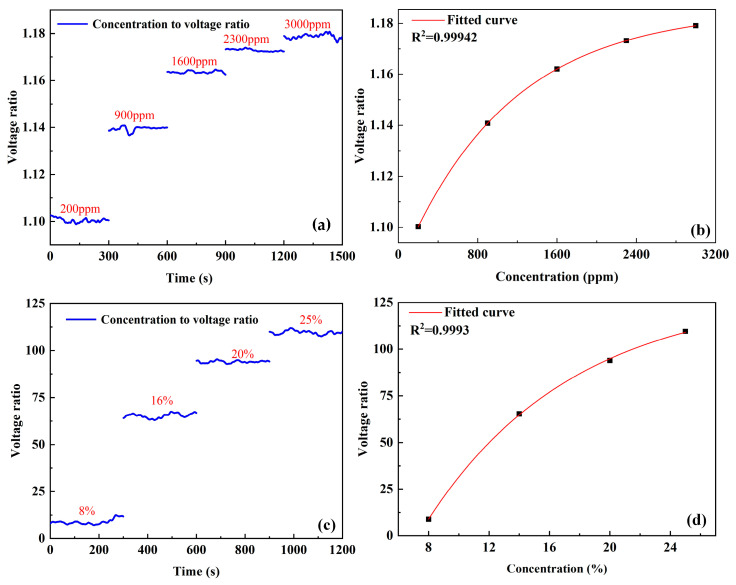
(**a**) Relationship between the first harmonic amplitude and measurement time for low concentration range. (**b**) Relationship between first harmonic amplitude and concentration for low concentration range. (**c**) Relationship between first harmonic amplitude and measurement time for high concentration range. (**d**) Relationship between first harmonic amplitude and concentration for high concentration range.

The sensor calibration is conducted under the condition of 20 °C temperature and 30% relative humidity. For the low concentration range of 200–3000 ppm, CO_2_ target gas samples with concentrations of 200 ppm, 900 ppm, 1600 ppm, 2300 ppm, and 3000 ppm were used for sensor calibration. For the high concentration range of 8–25%, CO_2_ samples with concentration levels of 8%, 16%, 20%, and 25% were adopted for calibration. Once the gas sample is injected into the chamber, the voltage signal output($\frac{U_{4}}{U_{2}}$) by the conditioning circuit is extracted using a software-based lock-in amplifier to obtain the first harmonic amplitude *U*. Sliding average filtering is performed on the first harmonic amplitude, *U*, which is then transmitted to the computer via a serial port. Each gas sample is tested for five minutes, and the relationship between the measured first harmonic amplitude *U* and measurement time is shown in Figure 4a,c, respectively. The relationship curves between the first harmonic amplitude *U* and gas concentration *C* are shown in Figure 4b,d, with the fitting equations as follows:

(1) low concentration range (200–3000 ppm):

(9) $$U=-0.10\times \exp[-\frac{C}{1084.16}]+1.18$$

(2) high concentration range (8–25%):

(10) $$U=-275.94\;\times \exp{[}^{-\frac{C}{9.72\;}}]+130.21$$

In Equations (9) and (10), *U* is the first harmonic amplitude of the two channels, and *C* is the gas concentration to be measured. The goodness of fit for the low concentration range is *R*^2^ = 0.99942, and for the high concentration range, it is *R*^2^ = 0.9993. From Equations (9) and (10), the gas concentration can be obtained as follows:

(1) low concentration range:

(11) $$C=1084.16\times \ln\frac{0.10}{1.18-U}C=1084.16\times \ln\frac{0.10}{U-1.18}$$

(2) high concentration range:

(12) $$C=9.72\;\times \;\ln\frac{275.94\;}{130.21-U}C=9.72\;\times \ln\frac{275.94\;}{U-130.21}$$

### 3.2. Stability and Allan Deviation Analysis

**Figure 5:**
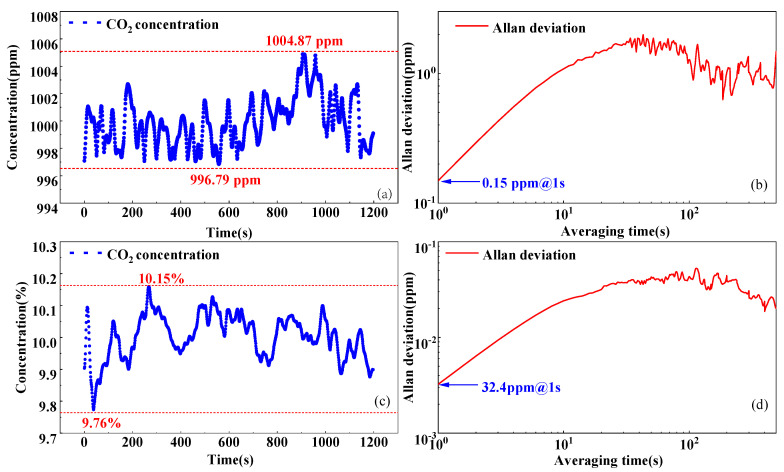
(**a**) Long-term measurement results of the 1000 ppm CO_2_ sample in low concentration range. (**b**) Allan deviation curve at low concentration range. (**c**) Long-term measurement results of the 10% CO_2_ sample in high concentration range. (**d**) Allan deviation curve at high concentration range.

To measure the stability and the limit of detection of the sensor in different measurement ranges, CO_2_ samples with concentration levels of 1000 ppm and 10% are pumped into the gas chamber for 20 min each. The measured first harmonic amplitudes are converted into gas concentration values in the corresponding ranges by Equations (11) and (12), respectively. The gas concentration changes are shown in Figure 5a,c. The measured gas concentration level ranges from 996.79 to 1004.87 ppm and 9.76 to 10.15%, respectively. The Allan deviation is used to calculate the limit of detection. As shown in Figure 5b,d, when the averaging time is 1 s, the limits of detection are 0.15 ppm and 32.4 ppm, respectively, for the two concentration ranges.

### 3.3. Temperature and Humidity Compensation

#### 3.3.1. Experimental Environment

**Figure 6:**
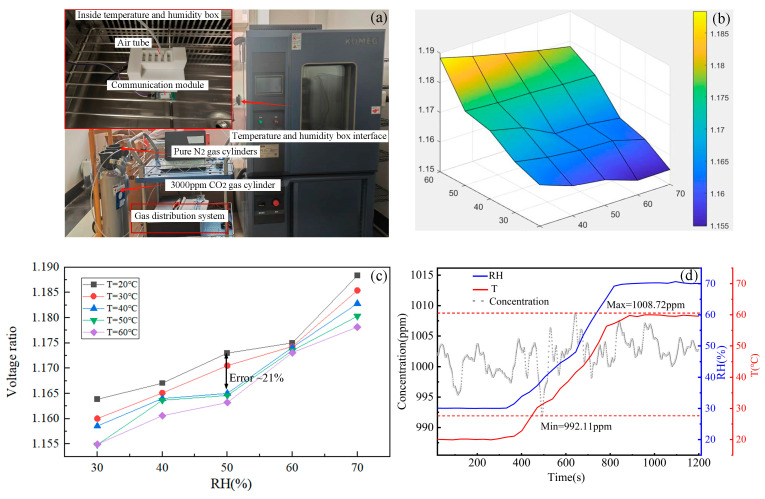
(**a**) A photo of the sensor temperature and humidity test. (**b**) The overall variation trend of the first harmonic amplitude under different temperatures and humidities. (**c**) The specific first harmonic amplitude data under different temperatures and humidities. (**d**) The measured gas concentration, as well as temperature and humidity, after compensation.

The environmental temperature and humidity are sampled using the SHT-31 sensor. For the sensor, the temperature measurement range is −40–125 °C, and the relative humidity (RH) measurement range is 0–100%. The temperature and humidity dependency of the sensor is conducted in a constant temperature and humidity chamber (Model: KMH-225S, KOMEG, Guangzhou, China, Alibaba Group). The adjustable temperature range is from −70° to 180 °C, and the adjustable humidity range is from 0% to 98%RH. A photo of the temperature–humidity test environment is depicted in Figure 6a. The gas sensor, fixed inside the chamber, is powered by the built-in lithium battery (Model: 18650, CKE, Shaoxing, China, Alibaba Group) and connected to the external computer via a WIFI module for test data transmission and collection. The gas sample is introduced into the chamber through a gas pipe. The test environmental parameters are summarized in Table 1. Once the environmental parameters reach the specified values and get stable, a five-minute temperature and humidity test experiment commences.

A total of 25 sets of experiments are carried out by setting different temperatures (20–60 °C) and different relative humidities (30–70%RH) and exposing the system to 1000 ppm CO_2_.

#### 3.3.2. Influence of Temperature and Humidity on the Sensor

The gas concentration to be measured is set at 1600 ppm. Initially, the humidity in the constant temperature and humidity chamber is set to 30%RH, with a temperature of 20 °C. Subsequently, the temperature is gradually increased to 60 °C while repeating the experiment. The humidity levels are adjusted with an increment of 10%RH, reaching up to 70%RH. Figure 6b illustrates the variation trend of the first harmonic amplitude of the sensor output under different temperature and humidity conditions. As depicted in Figure 6c, at identical temperatures, an increase in humidity results in light refraction through water vapor, leading to a reduction in light intensity. Consequently, the measured first harmonic amplitude gradually increases for the same gas concentration. Conversely, under constant humidity conditions, the measured first harmonic amplitude decreases gradually with rising temperature. The maximum error caused by temperature and humidity variation is about ~21%.

#### 3.3.3. Temperature and Humidity Compensation Algorithm

The optical path and circuit are affected by temperature and humidity fluctuations, resulting in signal drift and measurement error. Therefore, software is employed to compensate for the influence of environmental changes. A standard reference voltage value is established under the conditions of ambient temperature *T* = 20 °C, relative humidity *RH* = 60%, and CO_2_ concentration *C* = 1600 ppm. The difference between the absorption signal under different temperature–humidity conditions and that under the standard temperature–humidity environment are measured, and data fitting is performed. The influence of temperature and humidity on the first harmonic amplitude can be expressed as

(13) $$U_{\left(T,RH\right)}-U_{\left(20\;{}^{\circ}C,30\%\right)}=F_{\left(T,RH\right)}$$

Through fitting, the following equation is derived:

(14) $$F_{\left(T,RH\right)}=\frac{-0.01396+(-9.76E-4)\times T+0.00125\times RH+(-1.62E-5)\times RH^{2}+(1.88E-5)\times T\times RH}{1+(0.014)\times T+(0.00641)\times RH+(2.00E-4)\times T^{2}+(-3.26E-4)\times RH^{2}+(-1.30E-4)\times T\times RH}$$

Substituting Equation (14) into Equation (11), the gas concentration after temperature and humidity compensation can be achieved, as follows:

(15) $$C=1084.16\times \ln\frac{0.10}{{1.18-U}_{\left(T,RH\right)}+F_{\left(T,RH\right)}}$$

**Figure 7:**
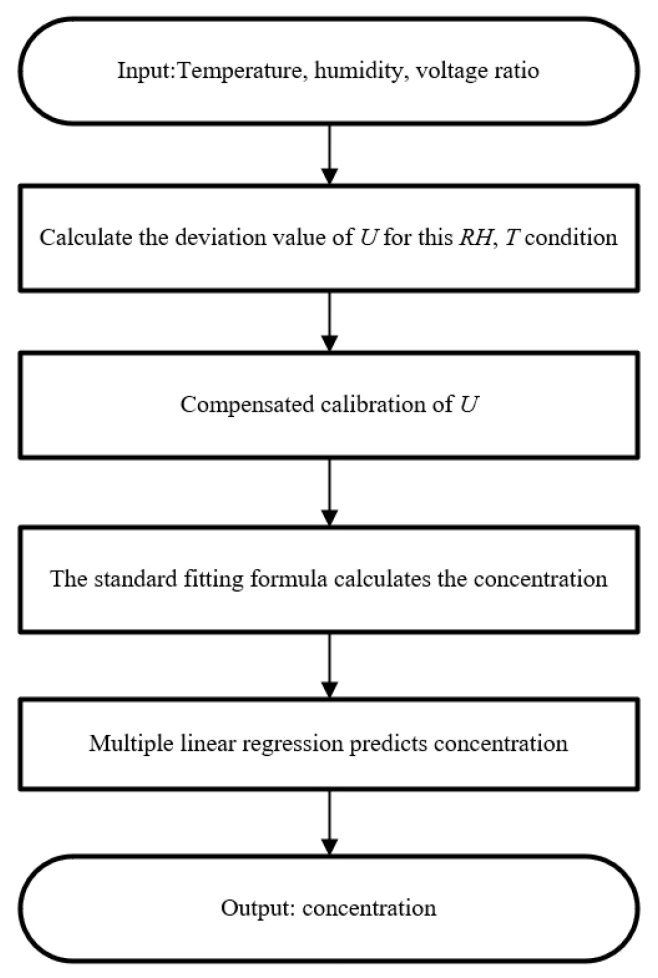
Flowchart of temperature and humidity compensation algorithm.

The flowchart of the temperature and humidity compensation algorithm is illustrated in Figure 7. The MCU transmits the collected first harmonic amplitude, temperature, and humidity data to the LabVIEW-based host computer. Subsequently, corresponding calculation and compensation are performed, and the resulting gas concentration is displayed on the computer. The constant temperature and humidity chamber is adjusted to 20 °C and 30%RH, respectively. After stabilizing the temperature and humidity, a 1000 ppm CO_2_ sample is injected into the gas cell, and then measurement is conducted for 400 s. Following this, the temperature and humidity are raised to 60 °C and 70%RH, and stable measurements are taken for another 400 s. As shown in Figure 6d, the measured CO_2_ concentration fluctuates between 992.11 and 1008.72 ppm, with a relative error of 0.87% after compensation. This demonstrates the effectiveness of the temperature and humidity compensation method.

## 4. Field Application of the Sensor System

### 4.1. Inspection Experiments

**Figure 8:**
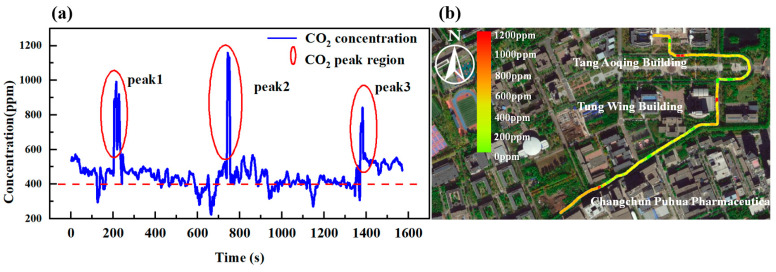
(**a**) Time measurement results of CO_2_ concentration. (**b**) Spatial distribution of measured CO_2_ concentration.

In outdoor inspection experiments, a global position system (GPS) module is mounted externally to facilitate communications to the host computer via a wireless communication module. The power source is provided with a 12 V lithium battery (Model: 18650, CKE). Figure 8a shows the continuous measurement results of CO_2_ concentration over time during mobile detection. Figure 8b shows the spatial distribution of CO_2_ concentration during the measurement process.

As shown in Figure 8a, three high-concentration areas were identified during the inspection. The first peak area, located at the front intersection of the Tang Aoqing Building, exhibits a peak concentration of 998.16 ppm, which can be attributed to the automobile exhaust emissions due to the high vehicle traffic in that area. The second area, positioned in front of the Dong Rong Building, recorded the highest concentration of 1157.59 ppm due to human exhalation caused by high pedestrian traffic during a local gathering activity. The third peak area, in the vicinity of Changchun Puhua Pharmaceutical Co., Ltd. (Changchun, China) showed a peak concentration of 841.73 ppm, associated with industrial CO_2_ emission from the pharmaceutical manufacturing processes.

### 4.2. Fire Detection Experiment

**Figure 9:**
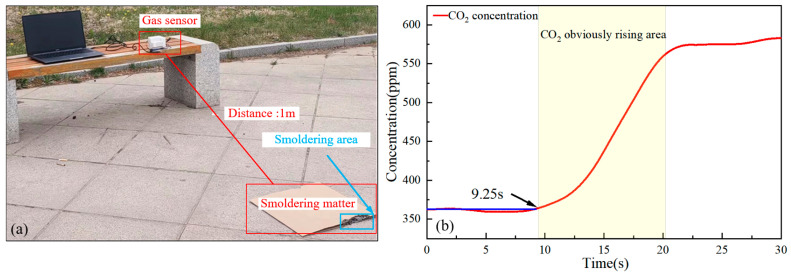
(**a**) Site of an experiment for smolder detection of fire. (**b**) CO_2_ concentration changes over time.

The fire from combustibles leads to a significant increase in CO_2_ concentration. As shown in Figure 9a, corrugated paper is placed about 1 m away from the sensor. After ignition, the gas concentration variation is recorded by the sensor, as illustrated in Figure 9b. Under outdoor conditions with a breeze, it is observed that the gas concentration starts to rise at 9.25 s after the corrugated paper is ignited, and the change in CO_2_ concentration is clearly detected. Real-time analysis of the change in CO_2_ concentration can enhance the efficiency of fire prevention and control, ultimately reducing fire risk.

### 4.3. Continuous Indoor CO_2_ Monitoring Experiment

**Figure 10:**
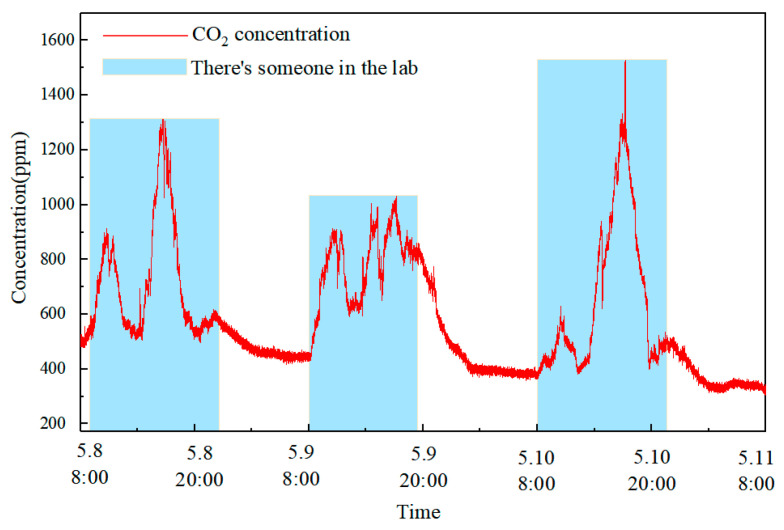
Long-term CO_2_ concentration detection results for three days.

When indoor air CO_2_ concentration becomes excessively high, it can adversely affect human health. Prolonged exposure to elevated CO_2_ levels may result in symptoms such as headaches, ringing in the ears, and elevated blood pressure. To verify the long-term stability of sensor monitoring, the CO_2_ sensor was placed in Laboratory 448, Block D, Tang Aoqing Building, Jilin University, and monitored continuously for 72 h (8 May to 11 May, 2024). The test results are presented in Figure 10. It is evident that the indoor CO_2_ concentration begins to rise from 8 am daily, with reductions observed during lunch and dinner periods. CO_2_ concentration is lowest at night due to a lack of human activity.

### 4.4. Underwater Carbon Dioxide Detection

**Figure 11:**
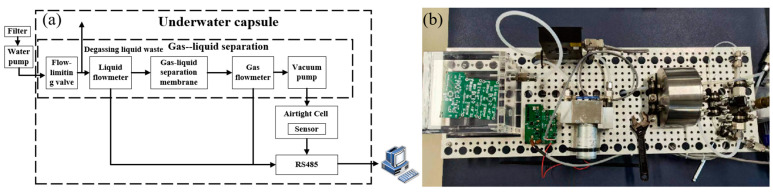
(**a**) Structure diagram of underwater CO_2_ detection system. (**b**) Physical diagram of underwater CO_2_ detection system.

Underwater carbon dioxide detection plays an indispensable role in various fields, including environmental protection, ecological research, marine science, and human health. As a critical indicator of water ecosystem health, underwater CO_2_ concentration not only reflects the balance between photosynthesis and respiration, which directly affects the survival and reproduction of aquatic organisms, but also serves as a key foundation for assessing the efficiency of the marine carbon cycle and understanding ecosystem dynamics. In the context of global climate change, the ocean, as the planet’s largest carbon sink, plays a crucial role in absorbing CO_2_ to mitigate global warming. Consequently, underwater CO_2_ monitoring has become essential for predicting changes in marine environments and addressing the challenges of ocean acidification. Additionally, in specific environments such as hot springs or underwater workplaces, detecting underwater CO_2_ levels is directly linked to the safety and health of personnel. The system’s structure and physical diagram are shown in Figure 11a.

The gas–liquid separation system consists of a metal chamber, a filter unit, a water pump (Model: SBE 5M Mini Submersible Pump, SEABIRD, Bellevue, WA, USA, Alibaba Group), a gas flowmeter (Model:FMA1700A, Dwyer Omega, Michigan City, IN, USA, Alibaba Group), a gas flowmeter (Model: FTB-1400, Dwyer Omega, Michigan City, IN, USA), a vacuum pump (Model: N84.3ANDC, KNF, Freiburg, Germany, Alibaba Group), and an aluminum alloy closed chamber. In measurement, the gas chamber is first filled with pure nitrogen and then pressurized to 0.4 atmospheres. Then, the pump begins cycling, utilizing the pressure difference and the hydrophobic selective permeability of the gas–liquid separation membrane to separate the gas from the water.

**Figure 12:**
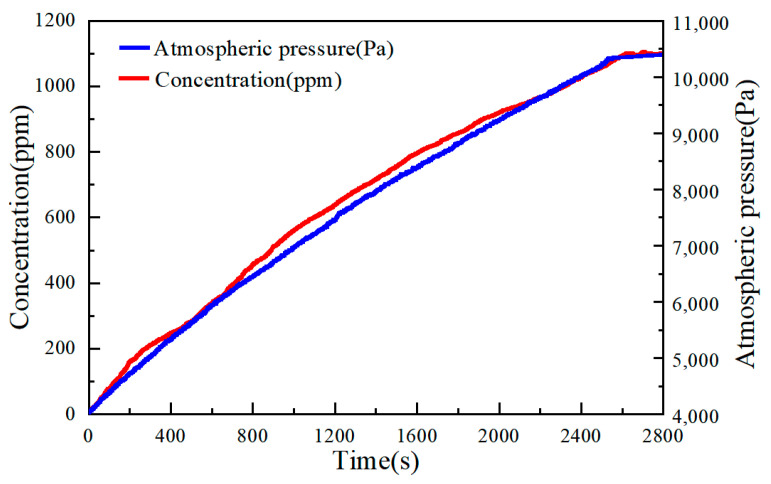
Underwater measurement results.

The underwater experiment of the system was conducted in the Yanhu area of Jilin University in September 2024, with the experimental results shown in Figure 12. As illustrated in the figure, initially, pure nitrogen gas was pumped into the closed air chamber, creating a negative pressure of 0.4 atmospheres. At this point, the carbon dioxide (CO_2_) data measured by the sensor were 0. As the gas–liquid separation device began to operate, gas was gradually separated from the water, causing the air pressure in the closed gas chamber to increase. This prompted the sensor to begin detecting CO_2_, and the concentration measured by the sensor rose in tandem with the increasing air pressure. After approximately 2400 s, the air pressure in the closed gas chamber reached a level approximately equal to the sum of the water pressure and atmospheric pressure. At this point, the gas–liquid separation device ceased operation, and the CO_2_ data stabilized. The gas concentration of CO_2_ in the separated gas from the lake water was approximately 1300 ppm. The experimental results demonstrate that the combined gas–liquid separation system effectively measures the carbon dioxide content in water.

## 5. Conclusions

A dual-range CO_2_ sensor system based on NDIR absorption spectroscopy is developed. The detection ranges of the system are designed to be 200–3000 ppm (low concentration range) and 8–25% (high concentration range). The sensor is calibrated with fitting goodness of *R*^2^ = 0.99942 for the low concentration range and *R*^2^ = 0.9993 for the high concentration range. Under environmental conditions of 20 °C temperature and 30% relative humidity, with an averaging time of 1 s, the detection limits are determined to be 0.15 ppm for the low range and 32.4 ppm for the high range. Compensation is implemented on the sensor to suppress the effects of temperature and humidity variations. The relative error after the compensation is recorded as 0.87%. Indoor and outdoor CO_2_ measurements are performed to validate the good characteristics of the sensor system.

## Figures and Tables

**Table 1:** Sensor test environment.

Relative Humidity (%)	30, 40, 50, 60, 70
Temperature (°C)	20, 30, 40, 50, 60

## Data Availability

No new data were created or analyzed in this study. Data sharing is not applicable to this article.
